# The influence of childhood adversities on mid to late cognitive function: From the perspective of life course

**DOI:** 10.1371/journal.pone.0256297

**Published:** 2021-08-16

**Authors:** Jing Ma, Yuanyuan Yang, Yang Wan, Chao Shen, Peiyuan Qiu

**Affiliations:** 1 Office of Cancer Prevention and Treatment, Sichuan Cancer Hospital & Institute, Sichuan Cancer Center, School of Medicine, University of Electronic Science and Technology of China, Chengdu, China; 2 The Brown School, Washington University in Saint Louis, Saint Louis, MO, United States of America; 3 West China School of Public Health and West China Fourth Hospital, Sichuan University, Chengdu, China; Montclair State University, UNITED STATES

## Abstract

**Background:**

The effects of childhood adversities on cognitive function in later life are well reported. However, few studies have examined the cumulative mechanism, especially in Chinese population. This study aims to explore this cumulative effects of childhood adversities on mid to late cognitive decline in China.

**Methods:**

Data were drawn from the second and third wave of the China Health and Retirement Longitudinal Study (CHARLS). We included 9,942 respondents aged 45 and above and retrospectively collected information on childhood adversities. Cognitive function was measured in three dimensions: orientation and calculation, immediate memory, and delayed memory. A structural equation model was employed for analysis.

**Results:**

Age (*β* = -0.155, *P*<0.001) and mid to late depressive symptoms (*β* = -0.041, *P*<0.001) showed direct effects on cognitive decline. Low mid to late life socioeconomic status (SES) showed a direct effect on mid-late cognitive impairment (*β* = 0.603, *P*<0.001) and an indirect effect through depression (*β* = 0.007, *P*<0.001). Low childhood SES (*β* = 0.310, *P*<0.001), lack of friends (*β* = 0.208, *P*<0.001), parental mental health problems (*β* = 0.008, *P*<0.001), and poor relationship with parents (*β* = 0.001, *P*<0.001) had an indirect effect on cognitive impairment.

**Conclusions:**

Childhood adversities had negative effects on cognitive function among middle aged and elderly population in China. The findings suggest that early counter measures on childhood adversities may lead to an effective reduction of cognitive impairment.

## Introduction

With the escalation of the aging population worldwide, cognitive impairment has emerged as a major public health concern [1,2]. Alzheimer’s Disease International (ADI) [3] estimated that by 2050 the number of people with Alzheimer’s disease (AD) globally would increase to 132 million from 47 million in 2015. China accounted for 25% of the world’s elderly patients with dementia in 2016, which has brought an immense socioeconomic burden [4]. It has been demonstrated that the annual cost of AD in China was over US $167 billion in 2015 and is projected to reach US $1.89 trillion by 2050, emphasizing the importance of dementia as a public health priority [5].

A sizable body of research have emphasized the importance of identifying the biological, psychological, and social factors for maintaining or improving cognitive function [6]. Age, educational attainment, family history, chronic diseases such as diabetes, mental health factors like depression, and repeated stress were major risk factors for cognitive decline [7–9]. Additionally, some studies indicated that adversities in early life such as an absent parent, bad early child–parent relationship quality, and inadequate social support were all negatively associated with cognitive capability in later life [10–14]. However, few studies have explored the mediating effects of childhood adversities on cognitive function in later life.

Life course theory was introduced to estimate the contribution of early life experiences to later life outcomes over the whole life process, which has provided a widely used framework for psychological and biological research [15]. Moreover, it provides significant insight into the study of aging and the accumulation of inequality.

Therefore, this study aimed to examine the cumulative effects of childhood adversities on cognitive impairment among middle aged and elderly Chinese people using the structural equation model (SEM).

### Theoretical framework for constructing the SEM

**Fig 1:**
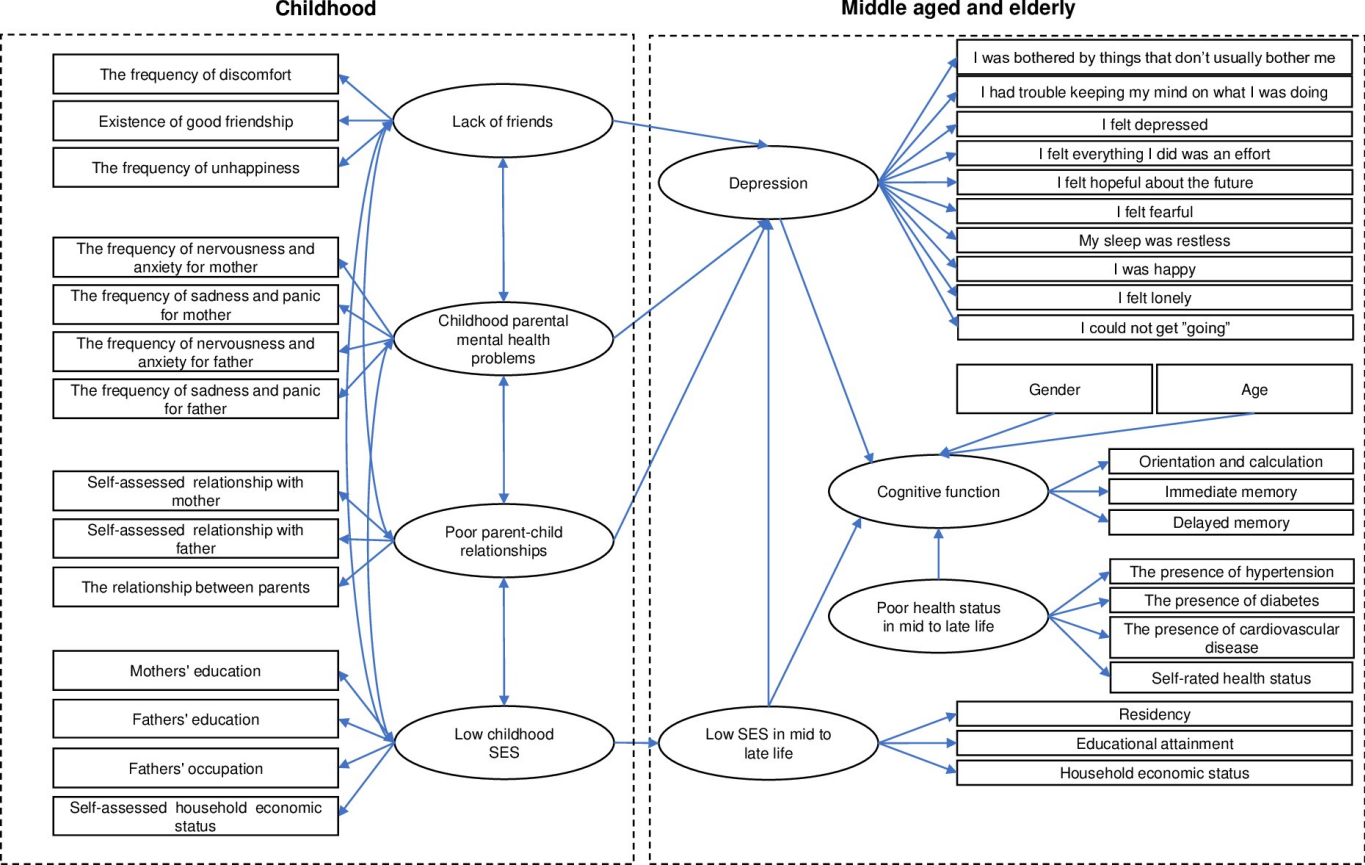
Initial structural equation model of the influence of childhood adversities on mid-late cognitive function.

Based on the framework of life course, we propose the following theoretical SEM model (Fig 1), which hypothesizes that four childhood adversities and other potential risk factors might act on mid to late life cognitive function in China.

#### Age, gender, and cognitive function

Age is regarded as the greatest risk factor for cognitive decline in mid to late life [16–19]. The percentage of people with Alzheimer’s increases dramatically with age: 3% for people aged 65–74, 17% for people aged 75–84, and 32% for people aged 85 or above [17]. In contrast, findings of a gender difference in cognitive deficit among middle aged and elderly populations remain controversial [20]. Some previous studies suggested gender differences in cognitive performance among Chinese older adults [21,22]. By contrast, other studies indicated non-significant direct association between gender and cognitive performance [23]. In our study, we intended to test the direct effects of age and gender on cognitive function in mid to late life.

#### Health status and cognitive function

Health status in middle aged and elderly people, such as chronic diseases, can also affect cognitive abilities. For example, several systematic reviews have shown an increased risk of cognitive decline among individuals with diabetes [24,25]. So, in our study we hypothesized that poor health status could directly affect cognitive function in mid to late life among the Chinese population.

#### Depression and cognitive function

Many studies have found that depression can adversely affect cognitive functioning in late life [26–30]. A recent longitudinal study showed that depressive symptoms increased cognitive decline in older adults [31], which is consistent with the findings of Chinese studies in which depression was generally associated with a certain degree of cognitive impairment [32,33]. Thus, we aimed to examine the direct effect of mid to late life depressive symptoms on cognitive impairment.

#### Socioeconomic status and cognitive function

A large number of studies have documented the impact of low SES in childhood on cognitive deficit in later life [34–36]. Lower SES has been found to be associated with a host of negative outcomes, including poorer general health, inequitable access to health services and increased risk for mental illness such as depression and anxiety [37–40]. Aartsen’s study further asserted that advantaged childhood SES was connected with higher cognitive functioning but stronger cognitive decline in older age [41]. A 5-year period cohort study also found that childhood SES is closely related to late-life baseline cognition [42]. Therefore, we intend to verify the direct and indirect association between low SES in mid to late life (associating it with low SES in childhood) and cognitive deficit mediated by depressive symptoms.

#### Childhood relationships with parents and cognitive function

Several studies reported the effect of suboptimal parent–child relationships on cognitive decline in mid to late life [14,43,44]. Adverse experiences from early life had a significant impact on individual outcomes in late life [45]. People who experienced childhood abuse were more likely to have trauma-associated symptoms such as personality disorders, substance abuse, posttraumatic stress disorder, chronic physical conditions, depression, and suicidal ideations [46–48]. Additionally, child neglect and domestic violence between parents have been shown to be associated with depression and health impairments [47,49,50]. Therefore, it is essential to test the potential connection between poor parent–child relationships and cognitive function indirectly through depression in our study.

#### Childhood parental mental health and cognitive function

Parental mental health problems were reported as important risk factors for cognitive functioning issues in children [51–53]. Familial factors in childhood such as shared genetic factors and a shared living environment had profound effects on children’s health outcomes [45]. For instance, Bennett’s study argued that the severity of mental health conditions for children aged 2–17 was positively related to parental mental illness [54]. Moreover, a 30-year-follow-up study found that children of parents with depressive symptoms were linked to a higher morbidity and mortality rate related to depression [55]. As such, we aimed to examine the indirect relationship between parental mental health issues and cognitive decline in their offspring through the mediation effect of late-life depressive symptoms.

#### Childhood friendship and cognitive function

Previous studies asserted that friendship support was a positive predictor for cognitive development [56–58]. Friendship is closely related to social adaptability, subjective well-being, and mental health. People with fewer friends were at a higher risk of suicide ideation, which was largely explained by self-assessed depression [59]. Teo’s research confirmed that high-quality social relationships were protective against depression [60]. An 18-year follow-up study also demonstrated that individuals with no friends were approximately twice as likely to experience internalizing symptoms (e.g. depression, anxiety, psychosomatic complaints) compared to those who had at least one friend in childhood [61]. Therefore, it is reasonable to test the association between lack of friends in childhood and mid to late life cognitive decline through depression in this study.

## Materials and methods

### Ethical approvals

The Ethics Review Committee of Peking University approved the study and informed consent was obtained from all participants. All methods were carried out in accordance with the relevant guidelines and regulations.

### Respondents

The data were derived from the second and third wave of the China Health and Retirement Longitudinal Study (CHARLS) (data and documentation are available at http://charls.pku.edu.cn/), which is a nationally representative longitudinal survey. CHARLS employed multistage probability sampling to recruit 150 counties of 28 provinces of mainland China except Hainan, Ningxia and Tibet. At the household level, CHARLS conducted mapping and operations within each village-level unit to create the sample frame. Therefore, households with a member 39 years of age or older were included. Then, randomly sampling was employed to recruit one Individual aged 39 years and over in the household. Selected individuals aged 45 years or older and their spouses were interviewed in the first wave of 2011, and those who were between 39 and 45 years of age were not interviewed and designated for inclusion in a future refreshment sample. More detailed information of the study design and sampling procedure can be found in the cohort profile of CHARLS [62].

The interviewers were trained at Peking University by CHARLS staff members. Data was collected in respondents’ homes by well-trained clinicians in a face-to-face, computer-aided personal interview (CAPI) manner. A total of 17,708 individuals agreed to participate in the baseline survey. The second wave of CHARLS, conducted in 2013, was a regular follow-up survey, which included demographic information, family structure, health status, income, and expenditures. In the second wave, 1,938 individuals were lost to follow-up, of which 431 were dead. Besides, 2,835 nonresponse sample and refresh sample in wave 1 were added for interview in wave 2. Totally, 18,605 individuals were surveyed in the second wave. The third wave survey, performed in 2014, was a special survey that retrospectively collected life history information of all longitudinal responsive samples. This wave included information regarding childhood SES, childhood history, health and health care history, and so forth. In the third wave, 2,134 individuals were lost to follow-up, of which 292 individuals were dead. And 4,072 refresh sample from wave 1 and non-response sample in wave 1 and wave 2 were included for interview in wave 3. Overall, 20,543 individuals were interviewed in the third wave.

**Fig 2:**
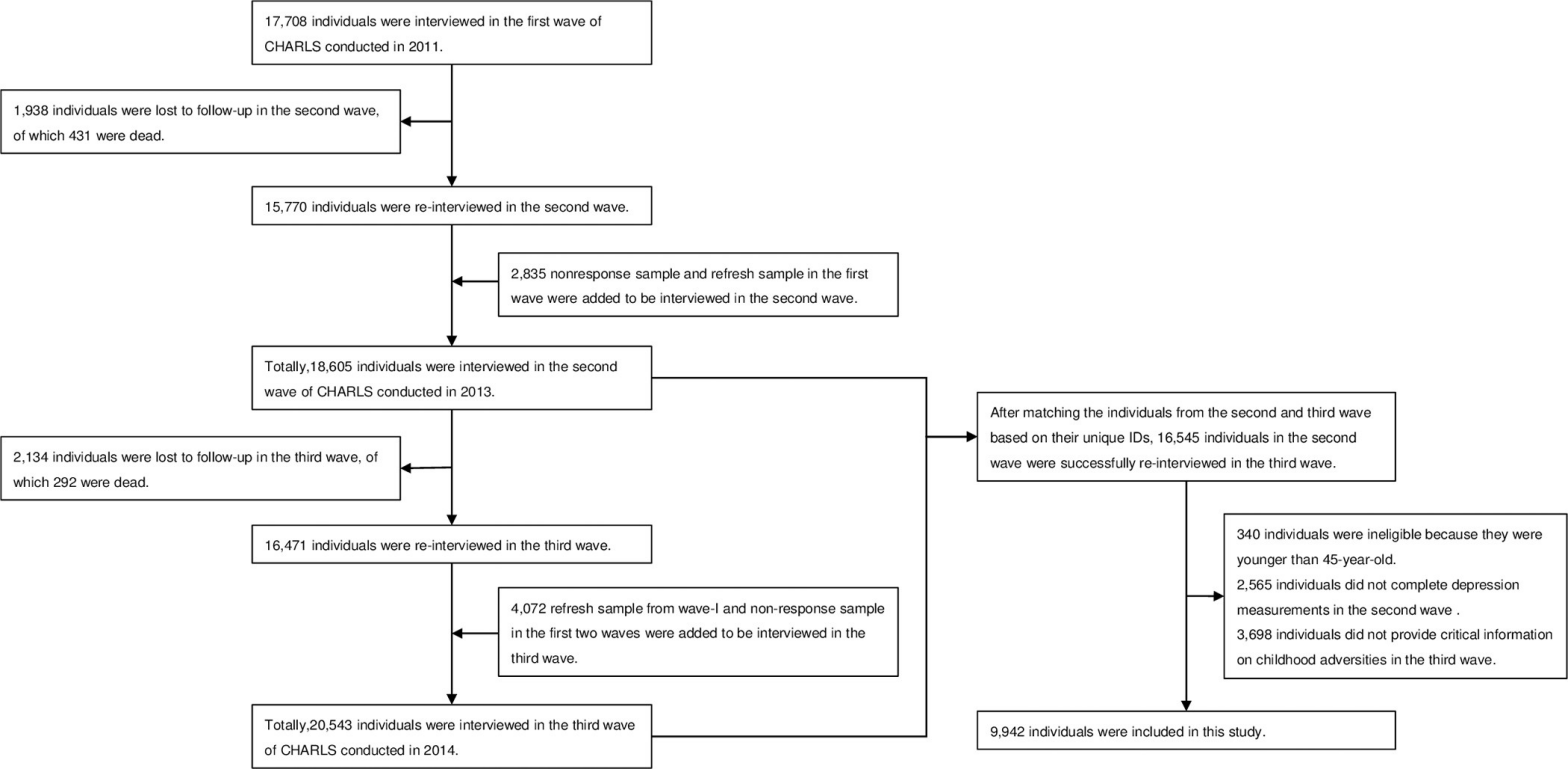
Flow chart of participants in the study.

We matched the individuals from Waves 2 and 3 based on their unique IDs in order to trace childhood adversities. The wave 3 survey successfully re-interviewed 16,545 individuals among the respondents in wave 2. To be eligible for the study, respondents had to satisfy three inclusion criteria: 1) they must have been aged 45 or older; 2) they fully provided critical information on childhood adversities and other potential risk factors; and 3) they were interviewed in both Waves 2 and 3. In the current analyses, 340 individuals were younger than 45-year-old, 2,565 individuals did not complete depression measurements in wave 2, and 3,698 individuals did not provide critical information on childhood adversities in wave 3. They were excluded. Thus, there were 9,942 individuals included in the final sample (Fig 2).

### Measures

#### Assessments of cognitive function

Cognitive function was measured in three dimensions (ten items for each dimension, 30 items in total), including orientation and calculation, immediate memory, and delayed memory (S1 Table). A 10-item questionnaire was adapted to assess orientation and calculation. It required respondents to tell exactly the current year, month, date, week, and season, and subtract 7 from 100 serially for five iterations. As for immediate memory assessment, participants were asked to immediately repeat in any order ten Chinese nouns just read to them. For assessment of delayed memory, respondents were required to recall the 10 words that had been read before by the investigators. A wrong answer for each item received a score of 0, and a correct answer got a score of 1. Adding up scores for each item generated a valid range from 0–30, with lower scores indicating a higher severity of cognitive impairment.

#### Assessments of depression

The 10-item Center for Epidemiological Studies Depression Scale (CESD-10) was deployed for the measurement of depressive symptoms [63,64]. It had 10 self-reported items, and responses to each item were rated on a 4-point Likert scale ranging from 0 (*rare)* to 3 (*most or all of the time)*. As for the frequency of negative emotions, the answers were rated from 0 (*rarely or none of the time*) to 3 (*most or all of the time*). Regarding the frequency of positive emotions such as “I was happy”, the score was reversely rated from 0 (*most or all of the time*) to 3 (*rarely or none of the time*). The sum of these 10 items (range: 0 to 30) reflected individuals’ depression. Thus, higher scores prompted worse depressive symptoms, and the cut-off point for depression was equal to or greater than 10 [64]. The Cronbach alpha coefficient of CESD-10 in this study was 0.797, consistently indicating comparable reliability with previous studies on depression among Chinese middle aged and older adults [65,66].

#### Assessments of socioeconomic status in mid to late life

SES in mid to late life was assessed by three indicators: residency, educational attainment, and household economic status. Residency was dichotomized into either an urban or a rural area. Educational attainment was categorized into six groups from illiteracy to bachelor’s degree or above. In most cases, household economic status refers to an income index such as family income. However, due to inaccurate income reporting in China, there are potential limitations when evaluating household economic status using income indicators. Thus, researchers proposed using an asset-based method in which they constructed an asset index to assess the household economic status [67,68]. In this study, we divided all households into five levels of “household economic status” based on the asset index we generated using principal component analysis on a scale from 1 (*very poor)* to 5 (*very good*) (S1 Table).

#### Assessments of poor health status in mid to late life

Health status in mid to late life was evaluated in four aspects: self-rated health status, the presence of hypertension, the presence of diabetes, and the presence of cardiovascular disease. The self-reported health status was assessed using a 5-point Likert scale from 1 (*very good*) to 5 (*very poor*). The remaining three items were dichotomous (S1 Table). People who self-reported having hypertension or diabetes, and who had objective measured values higher than the diagnostic standard were defined as having hypertension or diabetes. Cardiovascular diseases were self-rated by the participants.

#### Assessments of childhood adversities

*Low childhood socioeconomic status*. We measured childhood SES using parents’ education, father’s occupation, and self-assessed household economic status. Education level was categorized into six groups from illiteracy to bachelor’s degree or above. Father’s occupation was divided into nonagricultural, farming, and unemployment. In addition, a 5-point scale from 1 (*very poor*) to 5 (*very good*) was applied to estimate household economic status (S1 Table).

*Lack of friends*. Lack of friends was measured in three dimensions: the frequency of discomfort, the frequency of unhappiness, and existence of good friendship. The first two indicators used a 4-point Likert scale ranging from 1 (*never*), 2 (*not very often*), 3 (*sometimes*), to 4 (*often*). The last item was dichotomous (S1 Table).

*Childhood parental mental health problems*. We assessed parental mental health problems using the frequency of nervousness, the frequency of anxiety, the frequency of sadness, and panic for parents. All four indicators used a 4-point scale ranging from (*most of the time*) to 4 (*a little of the time*) (S1 Table).

*Poor parent–child relationships*. Parent–child relationships were evaluated by three indicators: a self-assessment of the mother–child relationship, a self-assessment of the father–child relationship, and the relationship between parents. All three indicators followed a 5-point Likert scale ranging from 1 (*poor*) to 5 (*excellent*) (S1 Table).

### Model construction and statistical method

Based on the theoretical framework, we constructed SEM to analyze the effect of childhood adversities on cognitive function among middle-aged and elderly Chinese individuals. Latent variables and observed variables in the model are shown in S1 Table. The initial structural equation model is shown in Fig 1.

Structural equation modelling analyses were performed with SPSS version 19.0 software (SPSS Inc., Chicago, IL, USA), using the weighted least squares means and variance adjusted estimation (WLSMV). The model was considered to have a good fit when root mean square error of approximation (RMSEA)<0.05 [69], comparative fit index (CFI)>0.90, the goodness of fit index (GFI)>0.90, and the normed fit index (NFI)>0.90 [70]. In the present study, the chi-square value was excluded when determining whether a structural equation model had a good fit or not as it is sensitive to sample size, and the chi-square value increased with a larger sample size. Adjusting or deleting the path between the two variables with lager modification index (MI) will be more conducive to the adjustment and optimization of the model. According to the model results, we reconstructed the model by removing non-significant associations and re-assessing the model fitness. Standardized regression coefficients (equivalent to path coefficients) among endogenous and exogenous latent variables were shown in the final model.

## Results

### Demographic characteristics

The description of respondents’ demographic characteristics is presented in Table 1. Of the 9,942 respondents, 52.9% were female. The average age was 59.93 (*SD* = 8.34), and only 1.86% of the respondents had an education level of some college or above. For location, 63.92% of participants lived in a rural area, and 81.78% reported fair or worse health status. Respondents with hypertension, diabetes, and cardiovascular diseases accounted for 38.74%, 8.32%, and 15.74% respectively. The score of depressive symptoms followed a skewed distribution: the median score was 6.00 (interquartile range, 4–11). The average cognitive function score was 13.53 (*SD* = 5.57). Other descriptive information about childhood adversities is shown in S2 Table. The differences of demographic characteristics between the excluded (n = 6,603) and included (n = 9,942) individuals were reported in S3 Table.

**Table 1:** Demographic characteristics of respondents (weighted).

	Overall (*N* = 9,942)
Variable	$\bar{x}$ ± *SD*/*n*(%)
Age, Mean	59.93±8.34
Age	
Aged 45–60	5543 (55.75)
Aged 60–75	3800 (38.22)
Aged 75–90	593 (5.96)
Aged 90+	6 (0.06)
Gender	
Male	4680 (47.07)
Female	5262 (52.93)
Educational attainment	
Illiterate	2488 (25.03)
Primary school	4058 (40.82)
Junior high school	2182 (21.95)
High school (secondary specialized school)	1029 (10.35)
Some college	133 (1.34)
Bachelor degree or above	52 (0.52)
Residency	
Rural	6355 (63.92)
Urban	3587 (36.08)
Household economic status	
Very poor	1821 (18.32)
Poor	1959 (19.70)
Fair	2035 (20.47)
Good	2051 (20.63)
Very good	2076 (20.88)
The presence of hypertension	
Yes	3852 (38.74)
No	6090 (61.26)
The presence of diabetes	
Yes	827 (8.32)
No	9115 (91.68)
The presence of cardiovascular disease	
Yes	1565 (15.74)
No	8377 (84.26)
Self-rated health status	
Very poor	1281 (12.88)
Poor	3571 (35.92)
Fair	3279 (32.98)
Good	1178 (11.85)
Very good	633 (6.37)
Cognitive function, Mean	13.53±5.57

### Structural equation modeling

**Fig 3:**
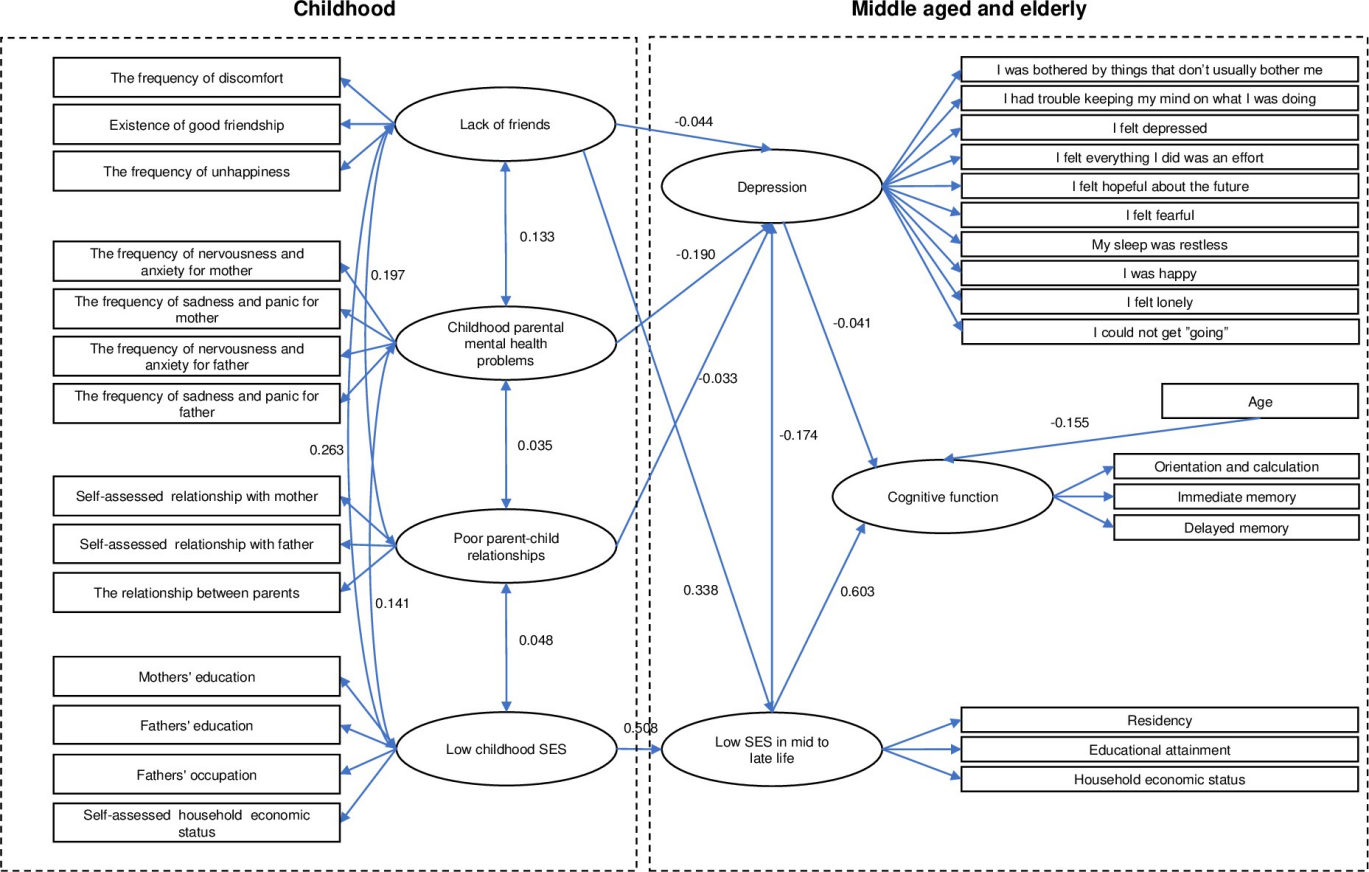
Final structural equation model of the influence of childhood adversities on mid to late cognitive function.

The confirmatory factor analysis for the measuring model based on the theoretical framework indicated an appropriate factor structure with a good model fit: *RMSEA* = 0.056, *GFI* = 0.904, *TLI* = 0.781, *CFI* = 0.799. All factor loadings from observed to latent variables were significant. Two hypothesized paths failed to reach significance: 1) from health status in mid to late life to cognitive function in mid to late life (*P* = 0.418) and 2) from gender to cognitive function in mid to late life (*P* = 0.062). Therefore, we deleted the insignificant pathways and reassessed each model. Then, new results indicated that the modification index (MI) between "lack of friends" and "socioeconomic status in mid to late life" was large (MI = 1417.749, parameter change = 0.569). After adding the pathway from "lack of friends" to" socioeconomic status in mid to late life", the final model was ascertained (Fig 3). A more satisfactory model was attained with a good model fit: *RMSEA* = 0.041, *GFI* = 0.952, *TLI* = 0.908, *CFI* = 0.918.

All path coefficients in the final model were standardized and significant (Table 2). Within the final model, lack of friends significantly indicated low SES in mid to late life (*β* = 0.338, *SE* = 0.014) and more severe depressive symptoms (*β* = -0.044, *SE* = 0.012). Low SES in mid to late life was strongly associated with higher severity of cognitive impairment (*β* = 0.603, *SE* = 0.051) and significantly predicted more severe depressive symptoms (*β* = -0.174, *SE* = 0.017). In addition, depression in mid to late life was significantly associated with cognitive impairment (*β* = -0.041 *SE* = 0.031). Parental mental health problems during childhood and bad parent–child relationships both had a significant influence on cognitive decline (*β* = -0.190, *SE* = 0.011; *β* = −0.033, *SE* = 0.012). We also found that increased age predicted a lower level of cognitive function (*β* = -0.155, *SE* = 0.028), and SES in childhood was strongly associated with SES in mid to late life (*β* = 0.508, *SE* = 0.081).

**Table 2:** Path coefficients and standard errors for the final SEM model (N = 9,942).

Independent variable	Dependent variable	*β* ^*a*^	*SE*	*CR*	*P*
Low childhood SES	Low SES in mid to late life	0.508	0.081	16.477	<0.001
Lack of friends	Low SES in mid to late life	0.338	0.014	18.799	<0.001
Lack of friends	Depression	-0.044	0.012	-2.719	0.007
Childhood parental mental health problems	Depression	-0.19	0.011	-15.765	<0.001
Low SES in mid to late life	Depression	-0.174	0.017	-9.954	<0.001
Poor parent–child relationships	Depression	-0.033	0.012	-2.789	0.005
Depression	Cognitive function	-0.041	0.031	-3.472	<0.001
Age	Cognitive function	-0.155	0.028	-15.107	<0.001
Low SES in mid to late life	Cognitive function	0.603	0.051	29.918	<0.001

SES, socioeconomic status; *β*, path coefficient; *SE*, standard error; *C*.*R*., critical ratio.

^a^, standardized parameter.

Standardized direct and indirect effects of childhood adversities on cognitive function in mid to late life are summarized in Table 3. It has been reported that SES in mid to late life has the largest effect on cognitive function (*β* = 0.610, *P*<0.001). Low SES in mid to late life had a positive effect on the cognitive impairment directly and indirectly, with the indirect effects mediated by depression. SES in childhood had the second largest total effect on mid to late life cognitive decline (*β* = 0.310, *P*<0.001) before the total effect of lack of friends during childhood (*β* = 0.208, *P*<0.001). Low SES in childhood had an indirect effect on poor cognitive performance mediated by low SES in mid to late life (*β* = 0.310, *P*<0.001). Lack of friends affected cognitive impairment through SES in mid to late life and depressive symptoms (*β* = 0.208, *P*<0.001). Parental mental health problems during childhood and poor parent–child relationships presented no direct effect on cognitive decline but contributed to depression (*β* = 0.008, *P*<0.001; β = 0.001, *P*<0.001). Additionally, age and depressive symptoms both indicated direct effects on cognitive function (*β* = -0.155, *P*<0.001; *β* = -0.041, *P*<0.001). The correlations of four latent variables of childhood adversities are demonstrated in Table 4.

**Table 3:** The standardized direct effects, indirect and total effects of the childhood adversities on cognitive function in mid to late life (*N = 9*,*942*).

Variable	Standardized direct effects	Standardized indirect effects	Standardized total effects
**Age**	-0.155^⁎⁎⁎^	-	-0.155^⁎⁎⁎^
**Low Childhood SES**	-	0.310^⁎⁎⁎^	0.310^⁎⁎⁎^
**Lack of friends**	-	0.208^⁎⁎⁎^	0.208^⁎⁎⁎^
**Childhood parental mental health problems**	-	0.008^⁎⁎⁎^	0.008^⁎⁎⁎^
**Poor parent–child relationships**	-	0.001^⁎⁎⁎^	0.001^⁎⁎⁎^
**Depression**	-0.041^⁎⁎⁎^	-	-0.041^⁎⁎⁎^
**Low SES in mid to late life**	0.603^⁎⁎⁎^	0.007^⁎⁎⁎^	0.610^⁎⁎⁎^

SES, socio-economic status.

^⁎⁎⁎^*P*<0.001.

**Table 4:** Correlations of four latent variables of childhood adversities (*N* = 9942).

Latent variables		*r* ^*a*^	*S*.*E*.	*C*.*R*.	*P*
Lack of friends	Childhood parental mental health problems	0.133	0.007	10.452	<0.001
Lack of friends	Poor parent–child relationships	0.197	0.007	14.845	<0.001
Lack of friends	Low childhood SES	0.263	0.005	12.24	<0.001
Childhood parental mental health problems	Poor parent–child relationships	0.035	0.005	3.075	0.002
Childhood parental mental health problems	Low childhood SES	0.141	0.003	8.558	<0.001
Poor parent–child relationships	Low childhood SES	0.048	0.002	3.399	<0.001

SES, socio-economic status; r, correlation coefficient; *SE*, standard error; *C*.*R*., critical ratio.

^a^, standardized parameter.

## Discussion

In our study, we intended to unpack the mechanism explanation between childhood adversities and cognitive impairment among middle aged and elderly Chinese people using different mediators. Consistent with our hypotheses and previous studies, age, depressive symptoms, and SES in mid to late life were directly associated with cognitive deficit [28,71,72]. Low SES in mid to late life showed the largest total effect on cognitive function among all the direct relationships, which is possibly due to reasons such as less access to health services, limited social support, fewer opportunities for success, and a higher probability of exposure to life adversities [40,73]. However, we did not find a significant direct association between gender and cognitive performance, which is consistent with the findings of a previous study in China [23].

It was shown that lack of friends in childhood indirectly affected cognitive decline in mid to late life mediated by depressive symptoms and low SES in mid to late life. Social support and social networks were protective factors for people’s mental health outcomes including cognitive ability in later life [74,75]. We argued that satisfying social relationships with others could enhance children’s psychological well-being by providing strong emotional support and strengthening coping abilities when children encounter life adversities. Additionally, friendship is closely related to parental educational level and occupation, which indirectly reflects SES [13]. Therefore, our results indicate that lack of friends was associated with an increasing risk of depressive symptoms and low SES in mid to late life, which subsequently increased the likelihood of low cognitive ability in mid to late life.

In accord with our hypothesis, it was shown that children whose parents had poor mental health were more likely to experience cognitive decline in later life, indirectly mediated through depressive symptoms in mid to late life. A recent study among older Chinese adults demonstrated that children who were exposed to parents with mental health problems were more likely to suffer depression in mid to late life [50]. Similarly, a significant association between depression and cognitive impairment in mid to late life was also found in our model, which is consistent with other findings [8,76,77]. A likely explanation could be that children living with parents who have poor mental health tend to receive inadequate emotional care and be influenced by substance abuse, anxiety, depression, suicide ideation, maltreatment, and violence from parents. These risk factors predict children’s mental health outcomes from childhood and have a cumulative impact on their depression and cognitive decline in mid to late life.

Our study also found a significant positive indirect association between poor parent–child relationships in childhood and a deficiency in cognitive function in mid to late life, which was mediated by depressive symptoms. A systematic review illustrated that children with secure attachments to parents tend to develop more positive health and mental health outcomes including better cognitive functioning [43]. Korkeila also found that good parent–child interactions were associated with increasing optimism, which could serve as a partial buffer when confronting adversities [78]. The indirect effects can be explained through the long-term impact of poor parent–child relationships developing over time on depression in mid to late life, which also significantly predicts cognitive impairment.

In addition, the results showed that experiencing low SES in childhood predicted a higher probability of cognitive decline, through the mediation effect of low SES in mid to late life as shown in previous studies [79,80]. Children growing up with low SES are less likely to gain adequate financial backups, higher educational attainment, and supportive social networks, which are crucial factors for future success. They have a higher risk of low SES in mid to late life, which in turn may increase their likelihood of experiencing poor nutrition and decrease their ability to afford health services such as prevention and treatments for depression.

Although this study benefitted from the use of life course theory to explore cumulative effects of childhood adversities on cognitive deficit in later life, we recognize several limitations as well. First, the CHARLS does not possess data from three provinces in mainland China (Hainan, Ningxia and Tibet), hence it is not entirely representative of the nation. Second, childhood information was collected with a retrospective method, which may cause recall bias. Third, as all the data were collected by questionnaire and were self-reported, it may have reporting bias. Fourth, individuals who had adverse events earlier in life may have died before reaching 45 years old, which may led to survival bias. Fifth, although the CHARLS survey was collected following a well-administered process with a low lost-to-follow-up rate, attrition bias and non-response bias may exist. Last, adverse events and other unobserved confounding factors were not available from the CHARLS, so we could not further test their effects on depression and cognitive impairment.

## Conclusions

This study examined the potential paths from four aspects of childhood adversities including lack of friends, poor parental mental health status, poor parent–child relationships, and low SES to cognitive impairment in middle-aged and elderly Chinese populations. The results demonstrated that all four variables were associated with mid to late life cognitive decline through indirect processes. From the life course perspective, the negative impacts of adverse experiences in childhood on people’s mental health are not isolated. Instead, these experiences cumulatively influence cognitive deficit in mid to late life. These important findings suggest the urgent need to invest available resources to prevent childhood adversities, subsequently reducing the prevalence of cognitive decline.

## Supporting information

S1 Table Assignment of latent variables and observed variables. (PDF)

S2 Table Description of childhood adversities (N = 9,942). (PDF)

S3 Table Demographic characteristics between the excluded and included individuals. (PDF)
