# Supplementary material for: The influence of childhood adversities on mid to late cognitive function: From the perspective of life course
Source: PLoS One. 2021 Aug 16;16(8):e0256297. doi: 10.1371/journal.pone.0256297 (PMC8366991; doi:10.1371/journal.pone.0256297)
Supplement: S1 Table — (PDF) [file pone.0256297.s001.pdf]

S1 Table. Assignment of latent variables and observed variables.

Latent variables	Observed variables	Assignment
	Gender	1=Male，0=Female
	Age	4=Above 90 years old，3=75~90 years old，2=60~75 years old， 1=45~60 years old
Low childhood socioeconomic status	Mother's education	5= Bachelor degree or above, 4= Some college, 3= High school (secondary specialized school), 2= Junior high school, 1= Primary school, 0= Illiterate
	Father's education	5= Bachelor degree or above, 4= Some college, 3= High school (secondary specialized school), 2= Junior high school, 1= Primary school, 0= Illiterate
	Father's occupation	2= Non-farming, 1= Farming, 0= Unemployment
	Self-assessed household economic status	5=Very good，4=Good，3=Fair，2=Poor ，1=Very poor
Lack of friends	The frequency of discomfort	4= Often, 3= Sometimes, 2= Not very often, 1= Never
	Existence of good friendship	2=No，1=Yes
	The frequency of unhappiness	4= Often, 3= Sometimes, 2= Not very often, 1= Never
Childhood parental mental health problems	The frequency of nervousness and anxiety for mother	4= A little of the time,3=Some of the time，2=Good part of the time，1=Most of the time
	The frequency of sadness and panic for mother	4= A little of the time,3=Some of the time，2=Good part of the time，1=Most of the time
	The frequency of nervousness and anxiety for father	4= A little of the time,3=Some of the time，2=Good part of the time，1=Most of the time
	The frequency of sadness and panic for father	4= A little of the time,3=Some of the time，2=Good part of the time，1=Most of the time
Poor parent-child relationships	Self-assessed relationship with mother	5=Excellent ，4=Very good，3= Good，2=Fair ，1=Poor
	Self-assessed relationship with father	5=Excellent ，4=Very good，3= Good，2=Fair ，1=Poor
	The relationship between parents	5=Excellent ，4=Very good，3= Good，2=Fair ，1=Poor
Poor health status in mid to late life	The presence of hypertension	1=Yes，0=No
	The presence of diabetes	1=Yes，0=No
	The presence of cardiovascular disease	1=Yes，0=No
	Self-rated health status	5=Very poor，4=Poor，3=Fair，2=Good ，1=Very Good
Depression	I was bothered by things that don’t usually bother me	3= Most or all of the time (5- 7 days)，2=Occasionally or a moderate amount of the time (3- 4 days)，1=Some or a little of the time(1- 2 days)，0=Rarely or none of the time(< 1 day)
	I had trouble keeping my mind on what I was doing	3= Most or all of the time (5- 7 days)，2=Occasionally or a moderate amount of the time (3- 4 days)，1=Some or a little of the time(1- 2 days)，0=Rarely or none of the time(< 1 day)

	I felt depressed	3= Most or all of the time (5- 7 days)，2=Occasionally or a moderate amount of the time (3- 4 days)，1=Some or a little of the time(1- 2 days)，0=Rarely or none of the time(< 1 day)
	I felt everything I did was an effort	3= Most or all of the time (5- 7 days)，2=Occasionally or a moderate amount of the time (3- 4 days)，1=Some or a little of the time(1- 2 days)，0=Rarely or none of the time(< 1 day)
	I felt hopeful about the future	3=Rarely or none of the time(< 1 day) ，2=Some or a little of the time(1- 2 days)，1=Occasionally or a moderate amount of the time (3- 4 days),0= Most or all of the time (5- 7 days)
	I felt fearful	3= Most or all of the time (5- 7 days)，2=Occasionally or a moderate amount of the time (3- 4 days)，1=Some or a little of the time(1- 2 days)，0=Rarely or none of the time(< 1 day)
	My sleep was restless	3= Most or all of the time (5- 7 days)，2=Occasionally or a moderate amount of the time (3- 4 days)，1=Some or a little of the time(1- 2 days)，0=Rarely or none of the time(< 1 day)
	I was happy	3=Rarely or none of the time(< 1 day) ，2=Some or a little of the time(1- 2 days)，1=Occasionally or a moderate amount of the time (3- 4 days),0= Most or all of the time (5- 7 days)
	I felt lonely	3= Most or all of the time (5- 7 days)，2=Occasionally or a moderate amount of the time (3- 4 days)，1=Some or a little of the time(1- 2 days)，0=Rarely or none of the time(< 1 day)
	I could not get ”going”	3= Most or all of the time (5- 7 days)，2=Occasionally or a moderate amount of the time (3- 4 days)，1=Some or a little of the time(1- 2 days)，0=Rarely or none of the time(< 1 day)
Low socioeconomic status in mid to late life	Residency	2= Urban, 1= Rural
	Educational attainment	5= Bachelor degree or above, 4= Some college, 3= High school (secondary specialized school), 2= Junior high school, 1= Primary school, 0= Illiterate
	Household economic status	5=Very good，4=Good，3=Fair，2=Poor ，1=Very poor
Cognitive function	Orientation and calculation	Continuous variable
	Immediate memory	Continuous variable
	Delayed memory	Continuous variable
