# Supplementary material for: The influence of childhood adversities on mid to late cognitive function: From the perspective of life course
Source: PLoS One. 2021 Aug 16;16(8):e0256297. doi: 10.1371/journal.pone.0256297 (PMC8366991; doi:10.1371/journal.pone.0256297)
Supplement: S2 Table — (PDF) [file pone.0256297.s002.pdf]

S2 Table. Description of childhood adversities (N=9,942).

Variable	n	%
LOW childhood socioeconomic status		
**Mother's education**		
Illiterate	8807	88.6
Primary school	959	9.6
Junior high school	114	1.1
High school (secondary Specialized school)	49	0.5
Some college	4	0
Bachelor degree or above	9	0.1
**Father's education**		
Illiterate	5914	59.5
Primary school	3283	33
Junior high school	442	4.4
High school (secondary Specialized school)	220	2.2
Some college	23	0.2
Bachelor degree or above	60	0.6
**Father's occupation**		
Non-farming	1783	17.9
Farming	8015	80.6
Not working	144	1.4
**Self-assessed household economic status**		
Very poor	104	1
Poor	800	8
Fair	5252	52.8
Good	1584	15.9
Very good	2202	22.1
Lack of friends		
**The frequency of discomfort**		
Never	1377	13.9
Not very often	880	8.9
Sometimes	1316	13.2
Often	6369	64.1

**Existence of good friendship**		
No	4855	48.8
Yes	5087	51.2
**The frequency of unhappiness**		
Never	1041	10.5
Not very often	738	7.4
Sometimes	2266	22.8
Often	5897	59.3
Childhood parental mental health problems		
**The frequency of nervousness and anxiety for mother**		
Most of the time	733	7.4
Good part of the time	914	9.2
Some of the time	1608	16.2
A little of the time	6687	67.3
**The frequency of sadness and panic for mother**		
Most of the time	544	5.5
Good part of the time	802	8.1
Some of the time	1488	15
A little of the time	7108	71.5
**The frequency of nervousness and anxiety for father**		
Most of the time	586	5.9
Good part of the time	827	8.3
Some of the time	1404	14.1
A little of the time	7125	71.7
**The frequency of sadness and panic for father**		
Most of the time	418	4.2
Good part of the time	612	6.2
Some of the time	1254	12.6
A little of the time	7658	77
Poor parent-child relationships		
**Self-assessed relationship with mother**		
Poor	86	0.9

Fair	1674	16.8
Good	1746	17.6
Very good	3098	31.2
Excellent	3338	33.6
**Self-assessed relationship with father**		
Poor	115	1.2
Fair	1870	18.8
Good	1843	18.5
Very good	3156	31.7
Excellent	2958	29.8
**The relationship between parents**		
Poor	192	1.9
Fair	2377	23.9
Good	1822	18.3
Very good	3209	32.3
Excellent	2342	23.6
