# Supplementary material for: The influence of childhood adversities on mid to late cognitive function: From the perspective of life course
Source: PLoS One. 2021 Aug 16;16(8):e0256297. doi: 10.1371/journal.pone.0256297 (PMC8366991; doi:10.1371/journal.pone.0256297)
Supplement: S3 Table — (PDF) [file pone.0256297.s003.pdf]

S3 Table. Demographic characteristics between the excluded and included individuals.

	**Included individuals (n=9,942)**	**Excluded individuals (n=6,603)**	**χ^2/t**	***P***
**Variable**	**$\bar{x}$ ± SD/ n(%)**	**$\bar{x}$ ± SD/ n(%)**		
Age，Mean	59.93±8.34	59.03±10.30	0.76	0.45
Gender			2.07	0.15
Male	4680（47.07)	3033（45.93)		
Female	5262（52.93)	3570（54.07)		
Educational attainment			10.04	0.07
Illiterate	2488（25.03)	1666（25.23)		
Primary school	4058（40.82)	2805（42.49)		
Junior high school	2182（21.95)	1375（20.83)		
High school (secondary specialized school)	1029（10.35)	618（9.36)		
Some college	133（1.34)	100（1.15)		
Bachelor degree or above	52（0.52)	38（0.58)		
Residency			3.51	0.06
Rural	6355（63.92)	4126（62.49)		
Urban	3587（36.08)	2477（37.51)		
The presence of hypertension			0.94	0.33
Yes	3852（38.74)	2608（39.50)		
No	6090（61.26)	3995（60.50)		
The presence of diabetes			0.84	0.36
Yes	827（8.32)	576（8.72)		
No	9115（91.68)	6027（91.28)		
The presence of cardiovascular disease			3.18	0.07
Yes	1565（15.74)	972（14.72)		
No	8377（84.26)	5631（85.28)		
